# Impact of Surgery on Older Patients Hospitalized With an Acute Abdomen: Findings From the Older Persons Surgical Outcome Collaborative

**DOI:** 10.3389/fsurg.2020.583653

**Published:** 2020-11-16

**Authors:** Hui Sian Tay, Adrian D. Wood, Ben Carter, Lyndsay Pearce, Kathryn McCarthy, Michael J. Stechman, Phyo K. Myint, Jonathan Hewitt

**Affiliations:** ^1^Department of Geriatric Medicine, Aberdeen Royal Infirmary, Aberdeen, United Kingdom; ^2^Institute of Applied Health Sciences, University of Aberdeen, Aberdeen, United Kingdom; ^3^Department of Biostatistics and Health Informatics, King's College, London, United Kingdom; ^4^Department of General Surgery, Manchester Royal Infirmary, Manchester, United Kingdom; ^5^Department of General Surgery, North Bristol National Health Service (NHS) Trust, Bristol, United Kingdom; ^6^Department of General Surgery, University Hospital of Wales, Cardiff, United Kingdom; ^7^Department of Geriatric Medicine, Cardiff University, Cardiff, United Kingdom

**Keywords:** operative management, conservation management, outcomes, older people, length of stay

## Abstract

**Background:** The impact of surgery compared to non-surgical management of older general surgical patients is not well researched.

**Methods:** We examined the association between management and adverse outcomes in a cohort of emergency general surgery patients aged > 65 years. This multi-center study included 727 patients (mean+/-SD, 77.1 ± 8.2 years, 54% female) admitted to five UK hospitals. Data were analyzed using multi-level crude and multivariable logistic regression. Outcomes were: mortality at Day 30 and 90, length of stay, and readmission within 30 days of discharge. Covariates assessed were management approach, age, sex, frailty, polypharmacy, anemia, and hypoalbuminemia.

**Results:** Approximately 25% of participants (*n* = 185) underwent emergency surgery. Frailty and albumin were associated with mortality at 30 (frailty OR = 3.52 [95% CI 1.66–7.49], albumin OR = 3.78 ([95% CI 1.53–9.31]), and 90 days post discharge (frailty OR = 3.20 [95% CI 1.86–5.51], albumin OR=3.25 [95% CI 1.70–6.19]) and readmission (frailty OR = 1.56 [95% CI (1.04–2.35)]). Surgically managed patients and frailty had increased odds of prolonged hospitalization (surgery OR = 5.69 [95% CI 3.67–8.80], frailty OR = 2.17 [95% CI 1.46–3.23]).

**Conclusion:** We found the impact of surgery on length of hospitalization in older surgical patients is substantial. Whether early comprehensive geriatric assessment and post-op rehabilitation would improve this outcome require further evaluation.

## Introduction

By 2030 the United States (US) population aged 65 and above will grow to 72 million (20% of the total population) from 40 million in 2010 (1). Older persons currently account for ~60% of the average general surgeon's workload, and up to 50% of all emergency surgical cases in the US (2–4). In the United Kingdom (UK), the second patient report of the National Emergency Laparotomy Audit (NELA) showed that almost half of the patients undergoing emergency laparotomy were over the age of 70 years (5). Surgical procedures performed in older persons with complicated medical needs have increased due to advances in surgical and anesthetics techniques, postoperative critical care, and the aging population (6, 7).

In a UK national database study by National Confidential Enquiry into Patient Outcome and Death (8), the overwhelming majority of perioperative deaths (86%) occurred in patients over the age of 60 years. Of these, 57% were after urgent or emergency surgeries (8). Higher likelihood of perioperative mortality in older age may relate to the greater physiological burden and greater risk for intra-operative complications associated with aging, multi-comorbidity, and frailty (5, 9, 10).

It is unclear whether patient characteristics are different between patients managed surgically or non-surgically. It is also currently unclear whether management approach is associated with adverse outcomes. Against this background, we aimed to determine the association between patient management and mortality at 30 and 90 days post admission, length of stay, and hospital readmission within 30 days of discharge in an unselected cohort of emergency general surgery patients aged over 65 years.

## Methods

### Study Design and Data Collection

In this UK-based multicenter retrospective analysis, consecutively admitted patients aged 65 years or over presenting to the acute general surgical unit of participating hospital sites throughout May and June 2013 and May and June 2014 were followed up for 90 days as part of the Older Persons Surgical Outcomes Collaboration (www.OPSOC.eu) (11, 12). The five participating sites were in Aberdeen, Bristol, Cardiff, Paisley and Manchester. All participating sites acquired relevant institutional approvals. As this study was deemed to be a service evaluation with data collected as part of routine patient care, there was no requirement for external ethical approval. Our study was conducted in accordance with the 1964 Helsinki declaration and its later amendments and complied with the data guardianship regulations governing the use of patient data in the United Kingdom (Caldecott principles). Research methods employed for this study comply with the STROCSS (Strengthening the Reporting of Cohort Studies in Surgery) guideline for observation studies (13).

### Study Covariates

Data collection methods have been previously described (11, 12). Briefly, baseline study data were collected from medical and nursing notes, prescribing charts and hospital electronic records by data collectors at individual sites according to study specific standard operating procedures. Information was collected on age, sex, medication use, frailty status, and presence of anemia and hypoalbuminemia (both common conditions in older age and potentially associated with unfavorable outcomes) on admission. Polypharmacy was defined as the use of five or more medications on admission. Medications started during hospital admission were not taken into account. Hemoglobin and albumin levels measured in routine bloods samples were used to determine presence of anemia (hemoglobin of 129 g/L or less) and hypoalbuminemia (albumin of 35 g/L or less). Frailty was measured using the Clinical Frailty Score, and categorized into patients non-frail (very fit, well, well with treated comorbid disease, apparently vulnerable), and those frail (mildly frail, moderately frail, severely frail) (14). Type of surgery (minor, intermediate, major minus, major plus, or complex major) was determined using the British United Provident Association (BUPA) schedule of procedures coding (15), which classifies common clinical procedures to facilitate administration of insurance claims (16). For this study, surgery type was categorized into two groups: major (major minus, major plus and complex major) and non-major (minor to intermediate). Non-surgically managed patients consisted both of persons who required emergency laparotomy but did not undergo surgery (e.g., due to poor fitness, unlikely to survive, advanced malignancy, patient decision) and persons in whom surgery was not indicated (e.g., uncomplicated diverticulitis, acute pancreatitis, per rectum bleeding, biliary colic).

### Study Outcomes

Data were collected via hospital records on mortality status at 30 and 90 days from the 1st day of hospital admission, length of stay, and readmission within 30 days of hospital discharge. All anonymized data were stored in a password-protected master file at the coordinating center in Cardiff. Missing data were assumed to be missing at random.

### Statistical Analysis

Analyses were conducted using Stata version 15. Descriptive statistics were determined by patient management approach (surgical vs. non-surgical and major vs. non-major surgery). Continuous variables are presented as mean (SD) values, and for categorical variables, the number and percentage are given. The association between management approach and study outcomes (mortality at 30 and 90 days post admission, length of hospitalization, and hospital readmission within 30 days of discharge) was examined using a multi-level crude and multivariable logistic regression, adjusting for age, sex, polypharmacy, frailty, anemia, and hypoalbuminemia. Sites were fitted with a random intercept model to account for heterogeneity between sites. A sensitivity analysis was carried out on day 30 readmission, after assuming all those that had died by Day 30 had been readmitted. Supplementary analyses in surgically managed patients examined the association between major surgery and study outcomes in similar multi-level crude and multivariable logistic regression models (with non-major surgery as the reference).

## Results

A total of 727 patients [mean age (SD) = 77.1 (8.2) years, 54% female] were included in this study, of whom 185 (25%) underwent emergency surgery (Table 1), and 214 (28.8%) were frail. Surgically treated patients were more likely to be male, and had lower prevalence of frailty, polypharmacy and anemia than those managed non-surgically. It is also suggested that few surgically managed patients were discharged before day 3 (9 cases, 4.9%), compared to those non-surgically managed patients (158 cases, 29.2%), and 144 (26.6%) non-surgical vs. 120 (64.9%) surgical participants had a length of stay >1 week. The majority (66.1%) of surgical cases were classed as major surgeries. Supplementary Table 1 shows participant characteristics by type of surgery.

**Table 1:** Participant characteristics by management approach.

**Characteristics**	**Non-surgical (*n* = 542)**	**Surgical (*n* = 185)**
Age, years	77.6 (8.4)	76 (7.7)
Sex, female	309 (57)	84 (45.4)
Frail	160 (29.5)	49 (26.5)
Polypharmacy	363 (67.7)	119 (65.7)
Hemoglobin <129 g/L	259 (52.2)	92 (49.7)
Albumin <35 g/L	252 (46.5)	93 (50.3)
**Outcomes**			
Mortality 30 days	25 (4.6)	5 (2.7)
Mortality 90 days	44 (8.3)	14 (7.7)
Length of stay: 1–2 days	158 (29.2)	9 (4.9)
3–6 days	240 (44.3)	56 (30.3)
7–13 days	95 (17.5)	55 (29.7)
>14 days	49 (17.5)	65 (35.1)
Readmission by Day 30	94 (17.7)	33 (18.2)

*Continuous data presented as mean (SD). All other data presented as n (%)*.

In a Crude unadjusted analysis both frailty and albumin were associated with increased odds of day 30 mortality (frailty OR = 3.52 [95% CI 1.66–7.49], albumin OR = 3.78 ([95% CI 1.53–9.31], Table 2), as well as at Day 90 mortality (Frailty OR = 3.20 [95% CI 1.86–5.51] and albumin OR = 3.25 [95% CI 1.70–6.19], Table 2). For patients that underwent surgery there was an association with an increase length of stay (>2-weeks), with crude OR = 5.69 (95% CI 3.67–8.80, Table 3), as well as association between those frail were associated with a longer stay OR = 2.17 (95% CI 1.46–3.23), and albumin OR = 1.61 (95% CI 1.09–2.38). Only patients that were frail were associated with an increased risk of readmission at Day 30, crude OR = 1.56 (95% CI 1.04–2.35). However, it should be remembered that patients who had died were excluded from this analysis, which would systematically bias these findings by their exclusion.

**Table 2:** Predictive factors for mortality at 30 and 90 days.

	**Mortality 30 days**				**Mortality 90 days**			
	**Crude OR (95% CI)**	***p*-value**	**Adjusted OR (95% CI)**	***p*-value**	**Crude OR 95% CI)**	***p*-value**	**Adjusted OR (95% CI)**	***p*-value**
Surgery	0.59 (0.22–1.58)	0.29	0.56 (0.20–1.52)	0.25	0.93 (0.49–1.74)	0.81	0.90 (0.46–1.73)	0.75
Age, under 80 years	- REF -				- REF -			
80–89 years	1.89 (0.89–4.00)	0.10	1.24 (0.55–2.82)	0.60	2.10 (1.20–3.68)	**0.009**	1.54 (0.84–2.82)	0.16
>90 years	1.04 (0.23–4.71)	0.96	0.58 (0.12–2.80)	0.50	1.68 (0.66–4.25)	0.27	0.83 (0.29–2.36)	0.73
Sex (reference female)	1.44 (0.70–2.97)	0.33	1.53 (0.70–3.32)	0.28	1.48 (0.87–2.53)	0.14	1.68 (0.95–2.97)	0.08
Frailty	**3.52 (1.66**–**7.49)**	**0.001**	**2.94 (1.28**–**6.76)**	**0.01**	**3.20 (1.86**–**5.51)**	**<0.001**	**2.67 (1.45**–**4.92)**	**0.002**
Polypharmacy ≥5 meds	2.04 (0.82–5.07)	0.12	1.39 (0.53–3.60)	0.50	1.39 (0.77–2.51)	0.28	0.96 (0.51–1.83)	0.93
Hemoglobin <129 g/L	1.81 (0.86–3.82)	0.12	1.12 (0.49–2.57)	0.78	1.81 (1.05–3.13)	**0.03**	1.19 (0.65–2.18)	0.57
Albumin <35 g/L	**3.78 (1.53**–**9.31)**	**0.004**	**3.04 (1.17–7.94)**	**0.02**	**3.25 (1.70–6.19)**	**<0.001**	**2.52 (1.26–5.3)**	**0.009**

*Data presented as OR (95% CI); bold values indicates P < 0.05*.

**Table 3:** Predictive factors for mortality at 30 and 90 days, prolonged hospitalization, and readmission 30 days after discharge.

	**Length of stay** **>** **2 weeks**				**Re-admission**			
	**Crude OR (95% CI)**	***p*-value**	**Adjusted OR (95% CI)**	***p*-value**	**Crude OR 95% CI)**	***p*-value**	**Adjusted OR (95% CI)**	***p*-value**
Surgery	**5.69 (3.67-8.80)**	**>0.001**	**6.60 (4.13-10.55)**	**>0.001**	1.04 (0.66-1.62)	0.87	1.00 (0.63-1.58)	1.00
Age, under 80 years	- REF -				- REF -			
80–89 years	**1.62 91.07–2.44)**	**0.02**	1.56 (0.97**–**2.51)	0.07	1.00 (0.65**–**1.53)	1.00	0.84 (0.53**–**1.32)	0.45
>90 years	0.86 (0.39–1.90)	0.71	0.70 (0.28–1.75)	0.44	0.93 (0.45–1.92)	0.83	0.73 (0.34–1.57)	0.43
Sex (reference female)	1.02 (0.69–1.50)	0.91	0.93 (0.60–1.56)	0.76	1.16 (0.79–1.70)	0.46	1.16 (0.78–1.74)	0.46
Frailty	**2.17 (1.46**–**3.23)**	**>0.001**	**2.54 (1.55**–**4.17)**	**>0.001**	**1.56 (1.04**–**2.35)**	**0.03**	**1.61 (1.02**–**2.54)**	**0.04**
Polypharmacy ≥5 meds	1.13 (0.75–1.72)	0.56	0.83 (0.51–1.34)	0.44	1.10 (0.72–1.67)	0.67	0.96 (0.62–1.49)	0.85
Hemoglobin <129 g/L	1.24 (0.84–1.82)	0.28	0.91 (0.57–1.46)	0.70	1.24 (0.85–1.83)	0.27	1.12 (0.74–1.71)	0.60
Albumin <35 g/L	**1.61 (1.09–2.38)**	**0.02**	1.45 (0.90–2.33)	0.13	1.41 (0.95–2.10)	0.09	1.32 (0.86–2.04)	0.21

*Data presented as OR (95% CI); bold values indicates P < 0.05*.

Multivariable models showed that management approach was not associated with patient mortality at 30 or 90 days from the 1st day of hospital admission (Table 2). Frailty and albumin were independently associated with mortality at day 30 and day 90 (Day 30 frailty adjusted OR = 2.94 [95% CI 1.28–6.76], albumin aOR = 3.04 [95% CI 1.17–7.94], Day 90 frailty aOR = 2.67 [95% CI 1.45–4.92], albumin aOR = 2.52 [95% CI 1.26–5.3]). After adjustment there was an association with a longer stay with surgery aOR = 6.60 (95% CI 4.13–10.55, Table 3), and frailty aOR = 2.54 [95% CI 1.55–4.17], but albumin was no longer found to associated OR = 1.45 [95% CI 0.90–2.33]. Consistently with the crude analysis, only frailty was associated with an increased odds of readmission aOR = 1.61 (95% CI 1.02–2.54, Table 3). Supplementary analysis shows that in surgically managed patients, type of surgery was not associated with 30 or 90 day mortality or readmission at Day 30 although major surgery was associated with increased odds for longer hospital stay [OR (95% CI) 5.35 (2.40–11.94), Supplementary Table 2].

## Discussion

In this UK-based multicenter observational study of consecutively admitted acute general surgical patients aged 65 years or over, those undergoing emergency surgery were more likely to be men with lower prevalence of frailty, polypharmacy and anemia. Surgical management was associated with longer hospital stay although management strategy was not related to mortality from the 1st day of hospital admission nor hospital readmission after discharge.

Frailty is present in at least 25% of the population aged over 85 years (16, 17). In our cohort, 28.7% of participants were categorized as frail according to the CSHA clinical frailty scale. Our findings showed that frailty is related to adverse outcomes are consistent with previous reports (18–25). Employing data from the Cardiovascular Health Study, it has been shown that frailty in participants over 65 years of age was independently predictive (over 3 years) of incident of falls, worsening mobility or activities of daily living, hospitalization, and death (19). As part of an American College of Surgeons quality improvement program dataset, frailty was assessed in over 30,000 emergency surgical patients aged over 60 years and was associated with increased 30-day mortality (22).

Hypoalbuminemia is present in ~20% of all acute medical admissions (26, 27). It is a marker of inflammation or malnutrition (28). It is well-recognized that hypoalbuminemia increases likelihood of poor outcomes such as mortality, morbidity, and prolonged hospital stay in acutely ill patients (29, 30). A meta-analysis of cohort studies and controlled trials revealed that a 10 g/L reduction in serum albumin results in 71% increase in odds of prolonged hospital stay (31). Our findings support these data. Addressing hypoalbuminemia in surgical patients may therefore be associated with better outcomes. This could potentially be achieved by better nutrition with high protein content peri-operatively (32). The European Society for Clinical Nutrition and Metabolism (ESPEN) guideline has recommended that all high risks and malnourished cancer patients undergoing major abdominal surgery should receive oral nutritional supplementations pre-operatively. Older persons with sarcopenia are considered to be high risk patients. (33).

Our data show that management approach does not impact on adverse outcomes in our general surgical patient cohort with the exception of prolonged length of stay in surgically managed patients. This is consistent with the UK National Emergency Laparotomy Audit which showed that length of stay after surgery increased with age (half of the patients under the age of 40 left hospital by day seven post-surgery, whereas half of the patients over the age of 80 were still in hospital after more than 14 days) (5). Older patients who underwent surgeries are more likely to develop post- operative complications, such as pneumonia, urinary tract infection, acute kidney injury, delirium, pain, functional decline and reduced mobility. They are more likely to need rehabilitation, care package, equipment to be put in their homes before discharged or discharge to a care home following surgery. Hence, older patients who undergo surgeries are likely to have longer length of stay.

A holistic approach with multidisciplinary team involvement has been shown to decrease length of hospital stay, intensive care admission, mortality, cost and hospital readmission (34–38). A prospective cohort study of patients aged 75 years or older examined acute tertiary hospital admissions to either the mobile acute care for the elderly (MACE) service (which composed of a geriatrician-hospitalist, fellow, nurse coordinator, and social worker) or the acute medicine service (usual care) (39). Patients managed by the MACE service were less likely to experience adverse events and had reduced odds for prolonged hospital stay when compared with patients receiving usual care (39). Provision of comprehensive geriatric assessment may therefore have a central role in optimizing the care and outcomes of older people in the acute surgical setting.

We found surgery in older people has major impact on length of stay among acute surgical admissions with > 5-fold increase in length of stay compared to their counterparts who received conservative management. Whilst this is perhaps not a fair comparison, this finding highlights the potential of prolonged hospitalization after surgery with deconditioning in older patients who are already frail.

Our study benefits from a number of strengths. We have conducted a multi-center study in different UK regions, thereby enhancing the generalizability of our results to the wider population. To the best of our knowledge, we are the first group to report on the relationship between management strategy (surgical or non- surgical) and adverse outcomes of mortality, prolonged hospitalization, and readmission following discharge in general surgical patients. We employed a validated and well-known scoring system to measure frailty. We acknowledge some limitations. Data were not collected on the diagnoses of the patients, whether surgeries were indicated, or if it was decided to manage patients non-surgically due to frailty or multi-morbidity. Our findings may be subject to potential bias from inter-observer variability, although the majority of our study parameters (polypharmacy, albumin, hemoglobin levels, age and gender) were objectively measured. Although all observers were trained in using the CSHA scale, we were not able to assess inter-rater variability.

A quarter of patients from our cohort had a surgery during their acute surgical admission in the UK setting. As the number of older adults who choose to undergo surgery continues to grow, it reinforces the need for optimal perioperative care to avoid complications in this at-risk population. Caring for older people with surgical emergencies is complicated and frailty, presence of co-morbidities, disease severity, alternative treatments and patients' wishes should be taken into consideration (40).

This study showed that management approach did not impact on adverse outcomes in our general surgical patient cohort with the exception of prolonged length of stay in surgically managed patients. We would encourage close working relationships with the allied health professionals to support early mobilization and rehabilitation for patients after emergency laparotomy. This has the potential to improve patient outcomes. Given the association between hypoalbunimaemia and increased odds of mortality in older persons admitted to hospital, the importance of pre-operative nutritional support with high protein content supplements should be prioritized (particularly in high risk patients such as those with sarcopenia). Thus, a holistic approach to the management of older persons hospitalized with an acute abdomen which includes comprehensive geriatric assessment and multidisciplinary team involvement may have the potential to optimize patient outcomes. Such an approach requires evaluation in future studies.

## Data Availability Statement

The raw data supporting the conclusions of this article will be made available by the authors, without undue reservation.

## Ethics Statement

Ethical review and approval was not required for the study on human participants in accordance with the local legislation and institutional requirements. Written informed consent for participation was not required for this study in accordance with the national legislation and the institutional requirements.

## Author Contributions

HT: data collection, literature review, and writing all versions of manuscript. AW: supervision, literature review, and writing all versions of manuscript. BC: statistics lead, supervision, co-ordination, and critical revision of the manuscript. LP, JH, and MS: data collection, supervision, and critical revision of the manuscript. KM: PI of OPSOC collaboration, data collection, supervision, and critical revision of the manuscript. PM: study conception, data collection, supervision, and critical revision of the manuscript. All authors involved in the interpretation of the results.

## Conflict of Interest

The authors declare that the research was conducted in the absence of any commercial or financial relationships that could be construed as a potential conflict of interest.
