# Supplementary material for: Impact of Surgery on Older Patients Hospitalized With an Acute Abdomen: Findings From the Older Persons Surgical Outcome Collaborative
Source: Front Surg. 2020 Nov 16;7:583653. doi: 10.3389/fsurg.2020.583653 (PMC7705344; doi:10.3389/fsurg.2020.583653)
Supplement: Supplementary file 1 [file Table_1.docx]

**Supplementary Table 1. Participant characteristics by type of surgery**

| Characteristics | Major (n=117) | Non-major (n=68) |
| --- | --- | --- |
| Age, years | 76.5 (7.2) | 75.2 (8.5) |
| Sex, female | 53 (45.3) | 31 (45.6) |
| Frail | 73 (62.9) | 38 (57.6) |
| Polypharmacy | 74 (64.9) | 45 (67.2) |
| Haemoglobin < 129 g/L | 80 (68.4) | 52 (76.5) |
| Albumin < 35g/L | 85 (72.6) | 52 (76.5) |
| **Outcomes** |  |  |
| Mortality 30 days | 3 (2.6) | 2 (2.9) |
| Mortality 90 days | 9 (7.8) | 5 (7.5) |
| Length of stay: 1 - 2 days | 0 (0) | 9 (13.6) |
| 3 – 6 days | 24 (20.5) | 25 (36.8) |
| 7 – 13 days | 37 (31.6) | 18 (26.5) |
| >14 days | 56 (47.9) | 16 (23.5) |
| Readmission by Day 30 | 17 (15.0) | 16 (23.5) |
