# Supplementary material for: Impact of Surgery on Older Patients Hospitalized With an Acute Abdomen: Findings From the Older Persons Surgical Outcome Collaborative
Source: Front Surg. 2020 Nov 16;7:583653. doi: 10.3389/fsurg.2020.583653 (PMC7705344; doi:10.3389/fsurg.2020.583653)
Supplement: Supplementary file 2 [file Table_2.docx]

**Supplementary Table 2. Association between major surgery and mortality at 30 and 90 days, prolonged hospitalisation, and readmission 30 days after discharge***

|  | Crude OR (95% CI) | *P* | Adjusted OR (95% CI) | *P* |
| --- | --- | --- | --- | --- |
| Mortality 30 days | 0.87 (0.14-5.33) | 0.88 | 0.55 (0.05-5.69) | 0.62 |
| Mortality 90 days | 1.05 (0.34-3.28) | 0.93 | 0.89 (0.25-3.21) | 0.86 |
| Length of stay > 2 weeks | **4.16 (2.03-8.52)** | **<0.001** | **5.35 (2.40-11.94)** | **<0.001** |
| Readmission after 30 days | 0.58 (0.27-1.23) | 0.16 | 0.60 (0.26-1.36) | 0.22 |

*Minor surgery was the reference category.
